# Acral Melanoma in Ethnic Lebanese Arab Patients: 12-Year Experience with a Rare Disease in the MENA Region

**DOI:** 10.3390/jcm14238320

**Published:** 2025-11-23

**Authors:** Nicole Charbel, Mohammad Hassan Hodroj, Mohamad Baqer Skaini, Ali Ghais, Maha Makki, Amal El Masri, Malak Ghezzawi, Joe Rizkallah, Lara Kreidieh, Jad Ibrahim, Firas Kreidieh

**Affiliations:** 1 Division of Hematology and Oncology, Department of Internal Medicine, American University of Beirut, Beirut P.O. Box 11-0236, Lebanon; nc47@aub.edu.lb (N.C.); mh309@aub.edu.lb (M.H.H.); ms400@aub.edu.lb (M.B.S.); aig08@mail.aub.edu (A.G.); axm09@mail.aub.edu (A.E.M.); lyk09@mail.aub.edu (L.K.); jai08@mail.aub.edu (J.I.); 2 Department of Internal Medicine, Clinical Research Institute, American University of Beirut, Beirut P.O. Box 11-0236, Lebanon; mm209@aub.edu.lb; 3 Department of Surgery, American University of Beirut, Beirut P.O. Box 11-0236, Lebanon; mg96@aub.edu.lb; 4 Department of Diagnostic Radiology, American University of Beirut, Beirut P.O. Box 11-0236, Lebanon; jr56@aub.edu.lb

**Keywords:** acral melanoma, melanoma, acral lentiginous melanoma, Middle Eastern, palmar, plantar, subungual, precision oncology

## Abstract

**Background/Objectives**: Acral melanoma (AM) is a rare and aggressive melanoma subtype that arises on sun-shielded, non-hair-bearing skin of the palms, soles, and nail beds. Although more common among individuals of non-European descent, AM remains underrecognized and understudied in the Middle East and North Africa (MENA). This study presents the first dedicated AM registry from Lebanon, aiming to characterize clinical, histopathological, and molecular features and evaluate diagnostic, referral, treatment approaches, and clinical outcomes over a 12-year period. **Methods**: This retrospective cohort study was conducted at the American University of Beirut Medical Center (AUBMC), a major tertiary referral center in the MENA region. All melanoma cases diagnosed between January 2012 and January 2024 were identified through electronic health records. From this cohort, all adult patients (≥18 years) with biopsy-confirmed AM or tumors located on the palms, soles, or under the nails were selected. **Results**: Our cohort consisted of 26 adult AM patients, identified from a total of 331 melanoma cases during the study period (8%). Median age at diagnosis was 58.5 years; 54% were female; and 96% of Middle Eastern origin. Most tumors were plantar (81%), and over half (53%) were diagnosed at early stages (Stage I–II). Surgery was performed in 92% of patients, yet 55% had positive margins. Sentinel lymph node biopsy was performed in 46%, and 35% received immunotherapy. Only 35% underwent molecular testing, identifying BRAF mutations in 11% of those tested; no patients received circulating tumor DNA analysis. At a median follow-up of 24.5 months, recurrence occurred in 27%, and metastasis developed in 23%. At the last follow-up, 92% were alive. **Conclusions**: Despite early-stage detection, high rates of positive margins and limited molecular testing reveal care gaps. This first national registry highlights the need to improve surgical management and expand access to precision oncology in the region.

## 1. Introduction

Melanoma constitutes around 20% of all skin cancer cases globally, and its incidence continues to rise. In 2022 alone, an estimated 331,647 new cases were reported worldwide [1,2]. The burden of melanoma varies by geographic location, with the highest incidence among fair-skinned populations, particularly in Australia/New Zealand, Western Europe, North America, and Northern Europe. In contrast, while Asian countries contribute to only 7.3% of all melanoma cases worldwide, they account for a disproportionate 21% of melanoma-related deaths, underscoring disparities in early detection, access to care, and outcomes. If 2020 trends continue, the global incidence of melanoma is projected to reach 510,000 cases and 96,000 deaths by 2040 [2]. A significant driver of this increase is the growing exposure of at-risk populations to ultraviolet (UV) radiation, which is a major and pervasive risk factor resulting from both natural sunlight and artificial sources [3]. This is further complicated by the complex interplay between the various other risk factors for melanoma, including phenotypic traits, UV exposure, and genetic susceptibility [4].

Among the various subtypes of melanoma, acral melanoma (AM), also known as acral lentiginous melanoma, stands out due to its unique clinical and epidemiological characteristics. AM appears on non-hair-bearing skin, such as the palms of the hand, soles of the feet, and under the nails [5,6]. Unlike other cutaneous melanoma subtypes associated with UV exposure, AM is not strongly linked with sun damage, fair skin, pre-existing melanocytic nevi, or a family history of melanoma [7,8]. Furthermore, AM exhibits distinct molecular features, including a lower tumor mutational burden and unique oncogenic driver mutations, setting it apart from other melanoma subtypes [9,10].

Despite advancements in melanoma treatment, patients with AM tend to have a poorer prognosis and a more aggressive phenotype. They carry a generally poor therapeutic response [11]. Although AM represents a smaller proportion of melanomas in fair-skinned populations, it accounts for a relatively higher percentage of cases among individuals of non-European ancestry. However, it remains underrepresented in global melanoma research [8].

Most available data on AM originate from European, North American, or East Asian cohorts, while patients from the Middle East and North Africa (MENA) are scarcely included or completely omitted [12]. To date, no dedicated Lebanese or regional registry has been published. As a result, region-specific patterns in diagnosis, histopathological characteristics, molecular testing, treatment, and outcomes remain poorly defined.

To address this gap, we present a 12-year experience from the American University of Beirut Medical Center (AUBMC), a leading tertiary referral center in the Middle East. We aimed to comprehensively characterize the clinical, histopathological, and molecular features of AM in Lebanon, evaluate referral patterns and diagnostic practices, and examine treatment modalities and patient outcomes.

## 2. Materials and Methods

This retrospective unicenter cohort study was conducted at AUBMC and was approved by the AUBMC Institutional Review Board (IRB) under protocol ID: BIO-2024-0302. All melanoma cases diagnosed between January 2012 and January 2024 were first identified using melanoma-related International Classification of Diseases (ICD) codes. After extraction, the list was manually screened to ensure accuracy: all non-melanoma skin cancers, including squamous cell carcinoma, basal cell carcinoma, and other lesions incorrectly captured under melanoma codes, as well as duplicate entries, were removed. This yielded a total of 331 confirmed melanoma cases, from which 26 adult patients (≥18 years) with biopsy-confirmed AM or tumors arising on the palms, soles, or nail apparatus were included. Patients whose lesions did not meet clinical or histopathologic criteria for AM were excluded.

Because several patients received their initial pathology diagnosis in external laboratories (public/governmental or private institutions), all outside pathology slides were re-reviewed at AUBMC by experienced pathologists and dermatopathologists to confirm the diagnosis prior to inclusion. Only cases validated by internal review were retained.

Baseline demographic and clinical data were extracted from electronic health records. At the time of diagnosis, the following information was collected: date of birth, date of diagnosis, age at diagnosis, gender, race, body mass index (BMI), Fitzpatrick skin type, sun exposure habits, coexistence of other skin cancers, history of dysplastic nevi, family history of melanoma, and financial coverage for treatment. Comorbidities were assessed using the Charlson Comorbidity Index (CCI), a validated tool for estimating mortality risk in longitudinal studies. One-year mortality rates for different scores were 12% for a score of 0, 26% for scores of 1–2, 52% for scores of 3–4, and 85% for scores of ≥5, supporting its utility in estimating the impact of comorbidities on patient outcomes [13].

Information on the referral process was also collected to assess potential diagnostic delays. This included the number of healthcare providers consulted before presenting to AUBMC and the type of healthcare provider who referred the patient to us.

Any documentation on disease extent and severity was also collected. Initial diagnosis information included the type of institution where the diagnosis was made, signs and symptoms at diagnosis, margins, histology of the tumor, presence of melanocytosis, tumor topography, Breslow thickness, Clark level, presence of ulceration, number of mitoses, pT classification, presence of regression, tumor-infiltrating lymphocytes (TILs) status, presence of lymphovascular invasion, presence of perineural invasion, diameter, tumor color, mutation status, circulating tumor DNA (ctDNA), lactate dehydrogenase (LDH) serum levels, nodal status, information about sentinel lymph node biopsy (SLNB), and the presence of metastasis at diagnosis (including metastasis staging and site if applicable). The mitotic rate was quantified by AUBMC dermatopathologists according to standardized institutional protocols, counted in the area of highest proliferative activity, and reported as mitoses per mm^2^ using contiguous 1-mm^2^ fields. Stage at diagnosis was documented using the 8th edition of the American Joint Committee on Cancer (AJCC) melanoma staging system [14].

Information was also collected at the last documented follow-up and throughout the course of the disease. Key variables included treatment modalities, recurrence, time to recurrence, development of metastasis, time to progression, and survival status.

The Statistical Package for the Social Sciences (SPSS) version 25.0 was used for data cleaning, management, and analysis (IBM SPSS Statistics for Windows, Version 25.0. United States of America). Descriptive statistics are expressed as median and interquartile range (IQR) for continuous variables and frequencies and percentages for categorical variables. In the bivariate analysis, the association between SLNB status and stage of diagnosis was carried out by using Fisher’s exact test. The correlation between two continuous variables (CCI, Breslow thickness, and time to recurrence) was assessed by using scatter plots and Spearman’s rank correlation coefficient. We also examined the association between recurrence and key clinical, histopathological, and genetic variables using Fisher’s exact test for categorical variables and the Mann–Whitney U test for continuous variables. A *p*-value < 0.05 was considered statistically significant.

## 3. Results

### 3.1. Patient Demographics

Twenty-six patients with an almost 1:1 male-to-female ratio (12 males, 14 females) were diagnosed with AM at AUBMC between 2012 and 2024. The median age at diagnosis was 58.5 years (IQR: 25.3), with ages ranging between 30 and 79. Most patients (96%) were of Middle Eastern descent, while only one patient was White non-Hispanic. The median body mass index (BMI) was 28.1 (IQR: 8.65). The median CCI score was 6.5 (IQR: 4.0; Table 1).

### 3.2. Referral Patterns

Assessment of the mechanism through which patients were referred to our hospital revealed that around half of the patients (46%) had consulted one healthcare provider prior to presenting to AUBMC, and 16% had consulted two providers, while 38% presented directly to us. Among those who initially sought care outside our institution, 73% were initially evaluated by a dermatologist, followed by 20% by other specialists, 13% by an oncologist, and 7% by a general practitioner/family medicine physician. Initial pathology diagnoses were distributed across AUBMC laboratory (40%), public/governmental laboratories (40%), and private local laboratories (20%; Table 2).

### 3.3. Signs and Symptoms at Diagnosis

Patients could present with more than one symptom, and the frequencies reflect all documented symptoms. The most frequently reported signs and symptoms at diagnosis included changes in lesion size (54%), bleeding or ulceration (42%), changes in color (33%), irregular borders (21%), and pain (21%; Table 3).

### 3.4. Tumor Characteristics

The most common tumor topography was plantar (81%), followed by subungual foot (15%). At diagnosis, over half (53%) were diagnosed at early stages (Stage I–II), with 38% having stage II and 15% having stage I disease at diagnosis. Only one patient (4%) had stage IV disease at diagnosis. SLNB was performed in 46% of cases, with 77% of patients being node negative. Positive margins were observed in 55% of patients. Melanocytosis was present in only 27% of patients whose melanocytosis status was reported.

Acral lentiginous melanoma was the most prevalent histological subtype (44%), followed by superficial spreading melanoma (20%). The median Breslow thickness was 3.9 mm (IQR: 4.45), and ulceration was observed in 55% of cases. Mitotic activity (≥1 mitosis) was present in 79% of patients. Most patients (53%) were classified as Clark level 4, followed by Clark level 3 (21%). pT classification was distributed across all categories, with T4 being the most frequent (38%). Regression was absent in all evaluated cases, although data were missing for 11 patients. Among patients with available TIL status, brisk TILs were identified in only 12%, with the remainder having non-brisk or sparse/absent TILs. Lymphovascular invasion was observed in one patient among the 17 evaluated. The median tumor diameter was 27.5 mm (IQR: 21.0), and most tumors were brown-pigmented (86%). Genetic testing was performed in 35% of patients, identifying BRAF mutations in only 11% of those tested. No patients underwent ctDNA analysis.

Two patients (8%) presented with metastasis at diagnosis: one had M1a disease (metastasis in the distant lymph nodes and skin), and one had M1b disease (metastasis in the lung; Table 4).

### 3.5. Treatment Modalities

Among the 26 patients included in this study, treatment modalities varied significantly, with surgery, immunotherapy, and chemotherapy being the primary treatments (Table 5).

- Surgery: At diagnosis, surgical interventions varied: 50% of patients underwent wide local excision (WLE) (31% with SLNB, 19% without SLNB), while 23% underwent amputation. Among the six patients who had amputation, only four (67%) were discussed in tumor boards. Excisional biopsy was performed in 19% of cases. Core biopsy was performed in 4% of patients, and punch biopsy in another 4%, as diagnostic procedures.
- Immunotherapy: Nine patients (35%) received immune checkpoint inhibitors (ICIs) during the course of their disease. Of these, four patients (15%) received nivolumab alone, one patient (4%) received ipilimumab alone, and four patients (15%) received a combination of ipilimumab and nivolumab. Additionally, three patients (12%) were treated with pembrolizumab alone. Notably, some patients received multiple types of immunotherapy sequentially.
- Among patients receiving ICIs, 33% had stage II disease, 44% had stage III, and 11% had stage IV.
- Chemotherapy: One patient (4%) received chemotherapy, which was paclitaxel.
- Other treatments: No patients received interferon or participated in vaccine trials.

### 3.6. Disease Course and Outcomes

The median time from diagnosis to the last follow-up was 24.5 months (IQR: 43.0). At the last follow-up, 92% of patients were alive. Recurrence occurred in 27% of patients, with a median time to recurrence of 6.33 months (IQR: 2.64).

Six patients (23%) who were not metastatic at diagnosis developed metastasis during the disease course, while 20 patients (77%) did not. The median time from diagnosis to progression was 19.13 months (IQR: 26.93). Specific metastatic sites included the skin (4 patients, 15%), lung (1 patient, 4%), brain (1 patient, 4%), liver (1 patient, 4%), and other sites (5 patients, 19%), with some patients experiencing metastases at multiple sites. The median LDH level at recurrence was 364.5 U/L (IQR: 829.5; Table 6).

### 3.7. Correlation Analyses

Among patients who underwent SLNB, no significant association was observed between the stage at diagnosis and SLNB status (*p* = 0.19).

The mean time to recurrence was 4.6 months for patients whose primary tumor was only managed by excisional biopsy, while it was higher at 6.33 months for those who underwent WLE + SLNB, 7.67 months for those who underwent amputation, and 7.82 months for those who underwent WLE without SLNB.

No significant correlation was identified between CCI and time to recurrence or progression (*p* = 0.32), nor between Breslow thickness and time to recurrence or progression (*p* = 0.23).

In the recurrence analysis, a significant association with recurrence was found for both the CCI (median 8.0 vs. 5.0; *p* = 0.03) and Breslow thickness (median 5.0 mm vs. 2.6 mm; *p* = 0.03). No significant differences were found for age, gender, LDH levels, ulceration, mitotic activity, stage at diagnosis, margin status, SLNB status, TILs, BRAF mutation, or immunotherapy use (all *p* > 0.05; Table 7).

## 4. Discussion

AM is the least frequent subtype among malignant melanomas. It was first described in 1970 and became a distinct histological subtype of melanoma in 1986. It accounts for approximately 2–3% of all melanomas in large U.S. and international cohorts [7,15]. In our cohort, AM represented 8% of diagnosed melanoma cases, which may reflect differences in population characteristics, where almost all of our patients were Middle Eastern. The median age in our cohort (58.5 years) was slightly younger than that reported in a large U.S. multicenter study (65 years). The sex distribution was comparable, with females representing 54% of our cases versus 53.3% in the U.S. cohort, yielding similar female-to-male ratios (1.17 vs. 1.14) [16].

Ethnic disparities in AM incidence and outcomes are well documented, with higher rates observed in non-European populations. An analysis of the Surveillance, Epidemiology, and End Results (SEER) database showed that underrepresented groups—including Black, Asian, Hispanic, and Pacific Islander patients—have lower melanoma-specific survival, a pattern that persists in AM even in the era of advanced therapies [10]. AM is also more frequently diagnosed at later stages, contributing to its poorer overall prognosis [15]. However, more than half of our patient population (53%) presented with early stage; while this observation may suggest earlier recognition in our setting, our retrospective design does not allow assessment of diagnostic timelines or population-level awareness.

In our cohort, recurrence was significantly associated with higher CCI scores and greater Breslow thickness. Prior research in acral lentiginous melanoma has similarly shown that increasing tumor thickness is an independent predictor of recurrence-free survival (HR = 1.13, 95% CI 1.07–1.20; *p* < 0.001) [16]. Although comorbidity burden has been less studied in this specific subtype, our finding linking higher CCI scores with recurrence is consistent with broader melanoma data showing worse prognosis with higher comorbidity burden [17].

Referral patterns in our cohort showed that 62% of patients consulted at least one healthcare provider before referral, with 46% seeing one provider and 16% seeing two. Most patients were initially evaluated by dermatologists (73%), followed by other specialists (20%), oncologists (13%), and general practitioners/family physicians (7%). In contrast, an Italian study reported that 58.1% of patients first consulted general practitioners, with dermatologists involved in 30.2% of cases and podiatrists in 9.3% [18]. The differences in referral patterns highlight variations in healthcare-seeking behavior and the role of primary care versus specialists in diagnosing AM. While dermatologists were more frequently involved early in our cohort, which may facilitate earlier recognition, definitive conclusions cannot be drawn from our data.

Despite acceptable survival at last follow-up, recurrence occurred in 27% of patients, and metastasis developed in 23% during the disease course. These findings emphasize the need for continued refinement of AM management.

Surgical management remains the cornerstone of AM treatment, including WLE with SLNB, amputation, or nodal dissection depending on stage and nodal involvement [19]. In our cohort, 92% of patients underwent surgery, although approaches varied, with only half receiving WLE. Several patients were managed with excisional, punch, or core biopsies, which are not recommended for definitive melanoma management because they may underestimate tumor depth and affect staging [20]. As WLE is the gold standard for primary cutaneous melanoma [21], these findings point to variability in adherence to surgical guidelines.

Tumor board involvement was inconsistent: among the six patients who underwent amputation, four were reviewed preoperatively, whereas two were not. As multidisciplinary evaluation is recommended for complex surgical decisions [22], ensuring more consistent tumor board participation may help standardize management and reduce practice variability.

Margin positivity was common, affecting 55% of patients. Although margin status was not associated with survival in our cohort, this may be related to missing data, limited sample size, or variability in surgical technique. National Comprehensive Cancer Network (NCCN) guidelines recommend margins based on Breslow thickness, ranging from 1 to 2 cm in AM [23], highlighting the importance of consistent adherence to established standards. While our sample size is small due to the rarity of AM, the 12-year timeframe improves the representativeness of the cohort within our setting.

Interferon was historically the only approved adjuvant therapy for melanoma, including AM, until the advent of ICIs, which have since reshaped the treatment landscape. Agents such as nivolumab, ipilimumab, and pembrolizumab have improved survival in advanced melanoma, particularly in combination regimens targeting PD-1 and CTLA-4 pathways, though with notable toxicity profiles [15]. However, several studies indicate that ICI efficacy in AM is lower than in cutaneous melanoma subtypes [24]. This reduced responsiveness may reflect biological features unique to AM; genomic and immune profiling by Carvalho et al. (2023) [10] showed lower tumor mutational burden, reduced neoantigen load, and frequent structural variations, all contributing to limited immunogenicity. These findings help explain the modest ICI response rates reported in the literature and are consistent with our cohort, in which only 35% of patients received immunotherapy. These observations support the need for treatment approaches that account for the distinct molecular and immune characteristics of AM, including potential combinations of ICIs with targeted or emerging immunomodulatory agents [10].

Evidence comparing ICIs and interferon specifically in AM remains limited, and no head-to-head studies exist [25]. Emerging data on targeted therapies—including c-Kit inhibitors such as imatinib, BRAF inhibitors, and CDK4/6 inhibitors—suggest possible activity in select patients, but these findings are derived from small studies and should be interpreted cautiously [26,27,28]. Larger, dedicated studies are needed to better define optimal systemic treatment strategies for AM.

In our study, none of the patients underwent ctDNA testing, despite its increasing relevance in advanced melanoma as a biomarker for prognosis, treatment response, and resistance monitoring [29,30]. Genetic testing was also limited, with only 35% of patients evaluated and BRAF mutations identified in 11% of those tested. European guidelines recommend mutation testing—particularly for BRAF V600—in advanced melanoma to guide personalized therapy [31]. Broader approaches such as next-generation sequencing (NGS) have shown greater utility in detecting actionable mutations than single-gene assays [32]. The limited use of ctDNA and NGS in our cohort suggests gaps in access to molecular diagnostics, which may reflect logistical or financial constraints. Expanding the availability of these tools could support more individualized management of AM in our setting.

Our study has several limitations. Its retrospective design relied on both conventional and electronic medical charts, which might have led to missing or incomplete information in some cases due to clarity or availability. The cohort spans 2012–2024, a period during which access to advanced treatments such as immunotherapy was inconsistent, limiting our ability to evaluate contemporary management patterns. Given the rarity of AM, the sample size was relatively small, which restricts statistical power, and findings should therefore be interpreted with caution. Although the 12-year duration improves case capture within our institution, generalizability remains limited, particularly because the vast majority of patients were of Middle Eastern descent, limiting comparisons across ethnic groups.

## 5. Conclusions

This 12-year AM retrospective registry, the first in Lebanon and the MENA region, highlights three main takeaways: suboptimal biopsy and surgical practices, limited access to molecular testing, and variability in management pathways. We also identified significant associations between recurrence and both CCI and Breslow thickness, underscoring the relevance of patient health status and tumor depth in outcomes. These findings support the development of a multicenter national registry to standardize care and improve early detection and treatment. Ongoing follow-up will be important as access to immunotherapy expands.

## Figures and Tables

**Table 1:** Demographics.

Characteristic	Value (N = 26)
Age at diagnosis (Years)	Median (IQR): 58.5 (25.3)
Sex	Male: 12 (46%)
	Female: 14 (54%)
Race	White, non-Hispanic: 1 (4%)
	Middle Eastern: 25 (96%)
Body Mass Index (BMI)	Median (IQR): 28.1 (8.65)
Charlson Comorbidity Index (CCI)	Median (IQR): 6.5 (4.0)

N = 26, total number of cases; IQR, interquartile range.

**Table 2:** Referral patterns.

Characteristic	Missing	Value (N = 26)
Number of healthcare providers seen before reporting to AUBMC	2	0: 9 (38%)
		1: 11 (46%)
		2: 4 (16%)
Type of healthcare provider referring patient to AUBMC	9	Dermatologist: 11 (73%)
		Oncologist: 2 (13%)
		General practitioner/family medicine doctor: 1 (7%)
		Other: 3 (20%)
Type of institution where the initial diagnosis was made	1	Public Laboratory: 10 (40%)
		Private Laboratory: 5 (20%)
		AUBMC Laboratory: 10 (40%)

N = 26, total number of cases; AUBMC, American University of Beirut Medical Center.

**Table 3:** Signs and symptoms at diagnosis.

Signs and Symptoms	n (%)
Size > 6 mm	2 (8%)
Bleeding/Ulceration	10 (42%)
Change in Size	13 (54%)
Change in Color	8 (33%)
Irregular Borders	5 (21%)
Inflammation	1 (4%)
Itching	2 (8%)
Pain	5 (21%)
Asymmetry	1 (4%)
Not Applicable	2 (8%)

Patients may present with more than one symptom. “Not Applicable” refers to cases with no documented symptoms.

**Table 4:** Tumor characteristics.

Characteristic	Missing	Value (N = 26)
Margins	4	Positive: 12 (55%)
Histology	1	Melanoma in situ: 3 (12%) Superficial spreading melanoma: 2 (20%) Nodular melanoma: 2 (8%) Acral lentiginous melanoma: 11 (44%) Other: 4 (16%)
Topography of tumor	0	Plantar: 21 (81%) Subungual (foot): 4 (15%) Subungual (hand): 1 (4%)
Breslow thickness (mm)	6	Median IQR: 3.90 (4.45)
Ulceration	6	Present: 11 (55%)
Melanocytosis	11	Present: 4 (27%)
Mitoses	7	0: 4 (21%) ≥1: 15 (79%)
Clark level	7	2: 2 (11%) 3: 4 (21%) 4: 10 (53%) 5: 3 (15%)
pT classification	2	Tis: 3 (12%) T1: 5 (21%) T2: 2 (8%) T3: 5 (21%) T4: 9 (38%)
Regression	11	Present: 0 (0%)
TILs status	10	Non-brisk: 7 (44%) Brisk: 2 (12%) Sparse/absent: 7 (44%)
Lymphovascular invasion	9	Present: 1 (6%)
Diameter (mm)	8	Median (IQR): 27.5 (21.0)
Tumor color	4	Yellow (amelanotic): 1 (5%) Brown (pigmented): 19 (86%) Mixed (partially pigmented): 2 (9%)
Genetic tests done	0	Yes: 9 (35%)
BRAF mutant	17	Yes: 1 (11%)
ctDNA	0	Tested: 0 (0%)
SLNB	0	Done: 12 (46%)
Nodal status	4	NX: 1 (5%) N0: 17 (77%) N1: 4 (18%)
Stage at diagnosis	0	Tis: 3 (12%) I: 4 (15%) II: 10 (38%) III: 6 (23%) IV: 1 (4%) Incomplete: 2 (8%)
Metastasis at diagnosis	1	Yes: 2 (8%)
Metastasis sites at diagnosis	1	M1a: 1 (4%) M1b: 1 (4%)
Serum LDH at diagnosis	15	Median (IQR): 183.0 U/L (45.0)

N = 26, total number of cases; IQR, interquartile range; mm, millimeters; Tis, melanoma in situ; T1–T4, pT classification stages; NX, regional lymph nodes cannot be assessed; N0, no regional lymph node metastasis; N1, metastasis in 1–3 regional lymph nodes; M1a, distant metastasis to lymph nodes and/or skin/subcutaneous tissues; M1b, distant metastasis to the lungs; LDH, lactate dehydrogenase; SLNB, sentinel lymph node biopsy; BRAF, B-Raf proto-oncogene gene mutation; ctDNA, circulating tumor DNA; TILs, tumor-infiltrating lymphocytes; Non-brisk, moderate immune response; Brisk, strong immune response; Sparse/Absent, minimal or no lymphocyte infiltration; U/L, units per liter.

**Table 5:** Treatment modalities.

Treatment Type	Value (N = 26)
Surgical procedures	WLE with SLNB: 8 (31%)
	WLE without SLNB: 5 (19%)
	Excisional biopsy: 5 (19%)
	Amputation: 6 (23%)
Core biopsy	1 (4%)
Punch biopsy	1 (4%)
Immunotherapy (ICIs)	Nivolumab alone: 4 (15%)
	Ipilimumab alone: 1 (4%)
	Ipilimumab/nivolumab combination: 4 (15%)
	Pembrolizumab alone: 3 (12%)
ICI use by stage	Stage II: 3 (33%)
	Stage III: 3 (45%)
	Stage IV: 1 (11%)
Chemotherapy	1 (4%)

N = 26, total number of cases; WLE, wide local excision; SLNB, sentinel lymph node biopsy; ICIs, immune checkpoint inhibitors.

**Table 6:** Disease course and outcomes.

Characteristic	Value (N = 26)
Status on last follow-up	Alive: 24 (92%) Dead: 2 (8%)
Time from diagnosis to last follow-up	Median (IQR): 24.5 (43.0)
Recurrence	Yes: 7 (27%)
Time from diagnosis to recurrence	Median (IQR): 6.33 (2.64)
Developed metastasis during the disease	Yes: 6 (23%)
Sites of metastasis during the disease	Skin: 4 (15%) Lung: 1 (4%) Brain: 1 (4%) Liver: 1 (4%) Other: 5 (19%)
Time from diagnosis to progression/metastasis	Median (IQR): 19.13 (26.93)
Serum LDH at recurrence	Median (IQR): 364.50 U/L (829.5)

N = 26, total number of cases; IQR, interquartile range; LDH, lactate dehydrogenase; U/L, units per liter.

**Table 7:** Associations between disease features and recurrence.

		Recurrence		*p*-Value
		No N = 19	Yes N = 7	
Gender	Male	10 (52.6%)	2 (28.6%)	0.39
	Female	9 (47.4%)	5 (71.4%)	
Age at diagnosis (years)	Median (IQR)	60.0 (26.0)	57.0 (16.0)	0.91
Serum LDH at diagnosis (U/L)	Median (IQR)	180.0 (106.5)	194.0 (-)	0.73
CCI	Median (IQR)	5.0 (5.0)	8.0 (1.0)	0.03
Breslow thickness (mm)	Median (IQR)	2.6 (4.2)	5.0 (5.3)	0.03
Ulceration	Absent	7 (46.7%)	2 (40.0%)	1.00
	Present	8 (53.3%)	3 (60.0%)	
Mitoses	0	4 (26.7%)	0 (0.0%)	0.78
	≥1	11 (73.3%)	4 (100.0%)	
Stage at diagnosis	Tis	3 (16.7%)	0 (0.0%)	0.83
	I-II	10 (55.6%)	4 (66.7%)	
	III–IV-Incomplete	6 (27.8%)	3 (33.3%)	
Margin status	Negative	7 (43.8%)	3 (50.0%)	1.00
	Positive	9 (56.3%)	3 (50.0%)	
SLNB performed	Not done	10 (52.6%)	4 (57.1%)	1.00
	Done	9 (47.4%)	3 (42.9%)	
TILs status	Non-brisk	5 (41.7%)	2 (50.0%)	1.00
	Brisk	2 (16.7%)	0 (0.0%)	
	Sparse/absent	5 (41.7%)	2 (50.0%)	
BRAF mutation	Yes	0 (0.0%)	1 (16.7%)	1.00
Immunotherapy use	Yes	2 (10.5%)	2 (28.6%)	0.29

Continuous variables are presented as median (interquartile range, IQR). Categorical variables are shown as frequencies and percentages. CCI: Charlson Comorbidity Index; LDH: lactate dehydrogenase; SLNB: sentinel lymph node biopsy; TILs: tumor-infiltrating lymphocytes.

## Data Availability

All data generated or analyzed during this study are included in this published article.
